# Multiple myeloma: clinical characteristics, current therapies and emerging innovative treatments targeting ribosome biogenesis dynamics

**DOI:** 10.1007/s10585-024-10305-2

**Published:** 2024-08-20

**Authors:** Mohamed H. Elbahoty, Bhavyasree Papineni, Rajeev S. Samant

**Affiliations:** 1 Department of Pathology, University of Alabama at Birmingham, Birmingham, AL USA; 2 Hematology Unit, Department of Internal Medicine, Faculty of Medicine, Alexandria University, Alexandria, Egypt; 3 Birmingham VA Medical Center, Birmingham, AL USA; 4 O’Neal Comprehensive Cancer Center, University of Alabama at Birmingham, Birmingham, AL USA; 5 WTI 320E, 1824 6th Ave South, Birmingham, AL 35294 USA

**Keywords:** Multiple myeloma, Treatment, Relapsed–refractory disease, Ribosome biogenesis, RNA polymerase I

## Abstract

Multiple myeloma (MM) is a clinical disorder characterized by aberrant plasma cell growth in the bone marrow microenvironment. Globally, the prevalence of MM has been steadily increasing at an alarming rate. In the United States, more than 30,000 cases will be diagnosed in 2024 and it accounts for about 2% of cancer diagnoses and more than 2% of cancer deaths, more than double the worldwide figure. Both symptomatic and active MM are distinguished by uncontrolled plasma cell growth, which results in severe renal impairment, anemia, hypercalcemia, and bone loss. Multiple drugs have been approved by the FDA and are now widely used in clinical practice for MM. Although triplet and quadruplet induction regimens, autologous stem cell transplantation (ASCT), and maintenance treatment are used, MM continues to be an incurable illness characterized by relapses that may occur at various phases of its progression. MM patients with frailty, extramedullary disease, plasma cell leukemia, central nervous system recurrence, functional high risk, and the elderly are among those with the greatest current unmet needs. The high cost of care is an additional challenge. MM cells are highly protein secretary cells and thus are dependent on the activation of certain translation pathways. MM also has a high chance of altering ribosomal protein-encoding genes like MYC mutation. In this article we discuss the importance of ribosome biogenesis in promoting MM and RNA polymerase I inhibition as an upcoming treatment with potential promise for MM patients.

**Graphical abstract:**

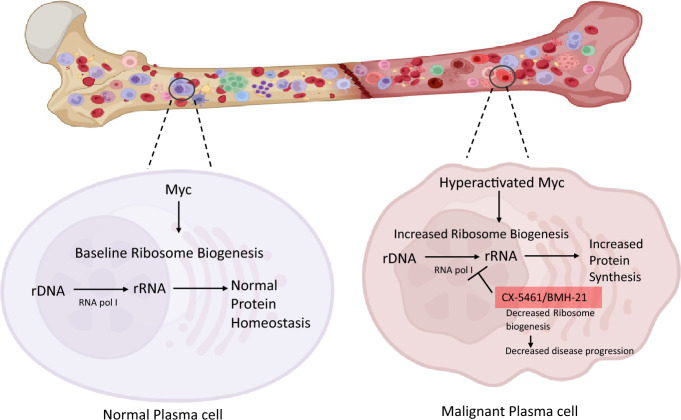

**Supplementary Information:**

The online version contains supplementary material available at 10.1007/s10585-024-10305-2.

## Introduction

Multiple myeloma (MM) is a pathological condition indicated by abnormal proliferation of plasma cells inside the bone marrow (BM) microenvironment. This syndrome is characterized by the presence of clonal proliferation of malignant plasma cells, the identification of monoclonal protein in the bloodstream or urine, and the onset of organ dysfunction. It comprises around 2% of the total neoplastic diseases and constitutes 13% of all hematologic malignancies [1, 2]. Globally, the frequency of MM has increased significantly, with a particular focus on its occurrence among men, those aged 50 and older, and populations living in high-income countries. The regions with the greatest incidence rates of MM include North America, Australia, New Zealand, and Europe, whereas the lowest rates are seen in Asia, excluding Western Asia. Around 37% of patients with first diagnosis of MM are below the age of 65, while 26% are within the age range of 65 to 74. The remaining 37% of patients are age 75 and above. Among individuals who are less than 30 years old, the prevalence of MM ranges from 0.02% to 0.3% [3, 4].

## Stages of multiple myeloma

Multiple myeloma (MM) is distinguished by substantial genetic abnormalities, resulting in a highly heterogeneous syndrome. This disorder progresses from a premalignant state known as monoclonal gammopathy of undetermined significance (MGUS) to a later stage known as smoldering multiple myeloma (SMM), which has the potential to proceed to symptomatic myeloma [5]. Monoclonal gammopathy of undetermined significance (MGUS) is a premalignant syndrome with absence of characteristic symptoms, which serves as a precursor for the development of MM. This disorder affects around 3% of individuals who are 50 years of age or older. Black individuals face a higher level of vulnerability compared to Whites [6]. MGUS has the potential to advance to MM or other associated myeloproliferative disorders and malignancies such as Waldenstrom macroglobulinemia, light chain amyloidosis and lymphoma at an annual rate of about 1%.

**Fig. 1:**
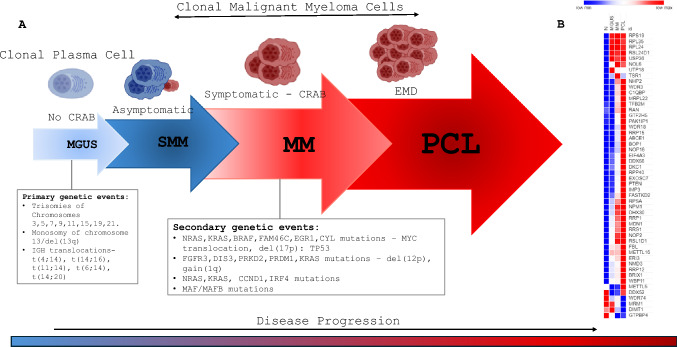
Multiple myeloma progression: this figure illustrates the clinical stages of multiple myeloma progression, beginning with monoclonal gammopathy of undetermined significance (MGUS), progressing through smoldering multiple myeloma (SMM), and culminating in symptomatic multiple myeloma (MM) and plasma cell leukemia (PCL). Key chromosomal abnormalities, including deletions, translocations, and their correlation with disease advancement and prognosis are indicated at each stage. **A** The schematic (left hand) is designed to aid understanding of the genetic landscape/milieu of multiple myeloma and its impact on clinical progression, potentially guiding therapeutic strategies and prognostic assessments. **B** The heatmap (right hand) displays differential gene expression associated with ribosome biogenesis across various stages of multiple myeloma, using GSEA to analyze data from the publicly available dataset GSE13591. Each column represents samples from a different disease stage: normal (N), monoclonal gammopathy of undetermined significance (MGUS), and plasma cell leukemia (PCL). Rows correspond to specific genes involved in ribosome biogenesis. Gene expression levels are color-coded, with red indicating higher expression and blue indicating lower expression. The analysis reveals a clear trend of increased expression of ribosome biogenesis genes as the disease progresses from MGUS and becomes most pronounced in PCL. This pattern suggests a potential role ribosome biogenesis in the progression and severity of MM. *Figures of cells* adapted from BioRender.com (2024). Retrieved from https://app.biorender.com/biorender-templates, *Heatmap* Morpheus, https://software.broadinstitute.org/morpheus. *GEO* Clough E, Barrett T (2016) The Gene Expression Omnibus Database. Methods Mol Biol 1418:93–110. *GSEA* (*analysis*) Subramanian et al. (2005)

Smoldering multiple myeloma (SMM) is a pathological condition that manifests as an intermediary stage between MGUS, and MM. It is characterized by the absence of clinical manifestations such as hyper**c**alcemia, **r**enal impairment, **a**nemia and **b**one lytic lesions (CRAB), despite the existence of monoclonal protein levels below 3 g/dL and/or aberrant plasma cells comprising 10–59% of the bone marrow [7]. It is considered as premalignant disease and exhibits a yearly risk of 10% for progressing into MM during the first 5 years after diagnosis. Over the subsequent 5 years the condition presents a 3% annual risk, and a 1.5% annual risk thereafter [8]. The unregulated proliferation of plasma cells is a defining feature of both active and asymptomatic MM that leads to CRAB. (Fig. 1A).

Multiple risk factors have been identified for the development of MM, including male gender, overweight status, occupational exposure to certain industries, and exposure to dioxin or Agent Orange. However, the precise etiology of MM remains elusive. Common findings in newly diagnosed individuals with MM include anemia, presence of one or more lytic lesions on conventional radiograph (79% of cases), elevated creatinine levels (19% of cases), lymphadenopathy (1% of cases), hypercalcemia (13% of cases), and thrombocytopenia (5% of cases) [9]. About 3% of newly diagnosed patients have extramedullary illness detected as extraosseous plasmacytomas on imaging, with central nervous system involvement, and plasma cell leukemia.

## Diagnosis of multiple myeloma

Diagnosis of MM is established by one or more myeloma defining events (MDE), as well as the presence of 10% or more clonal plasma cells in bone marrow or biopsy confirming plasmacytoma. It is characterized by a group of clinical symptoms (CRAB), with the presence of three biomarkers (Table 1). When there is a clinical suspicion of MM, a battery of tests to determine the presence of M proteins is advised. Serum protein electrophoresis (SPEP), serum immunofixation (SIFE), and the serum free light chain ratio (FLCR) testing should all be performed. It is advised that bone marrow exams be performed using fluorescent in situ hybridization (FISH) methods during the initial diagnosis procedure. The use of conventional karyotyping for the detection of hypodiploidy and deletion 13 is critical for risk stratification. If available, gene expression profiling (GEP) has the potential to give additional prognostic relevance. Low-dose whole-body computed tomography (WB-CT) or positron emission tomography/computed tomography (PET/CT) imaging methods are the most effective for determining the severity of bone disease. MRI scans have proven to be useful diagnostic tools in patients with suspected SMM [10].

**Table 1:** An overview of the current standard of care for multiple myeloma [10, 13–16]

Clinical stage	Stage characteristics	Standard protocol	New treatments	Clinical trials
MGUS	• Clonal BMPCs < 10% • Serum monoclonal protein < 3 g/dL • No CRAB	Close surveillance, frequent checks	N/A	• NCT00665652 (Lenalidomide) • NCT06046287 (Daratumumab) • NCT03236428 (Daratumumab)
SMM	• MCP should be present: serum (IgG or IgA) ≥ 3 g/dL, urine MCP ≥ 500 mg per 24 h, and/or clonal BMPCs10–60% • Neither amyloidosis nor MDE are present	*• Low risk* observation *• High risk* lenalidomide or lenalidomide plus dexamethasone (Rd) for 2 years, or enrollment in a clinical trial	N/A	• NCT03236428 (Daratumumab) • NCT00099047 (celecoxib) NCT02279394 (Elotuzumab, Lenalidomide, Dexamethasone) • NCT05597345 (Selinexor) • NCT02916771 (Ixazomib, Lenalidomide, Dexamethasone)
MM	• Bony or extramedullary plasmacytoma verified by biopsy or 10% or more clonal BMPCs • Any of the following may define myeloma: End organ damage: 1 Hypercalcemia 2 Kidney failure 3 Anemia 4 Single or several osteolytic bone lesions on radiograph scans 60% BMPCs Involvement of a serum FLCR of 100 or higher or a level of 100 mg/L or higher > 1 mm-sized MRI focal lesion	A-ASCT eligible: • 3–4 Cycles induction therapy. Triplet or quadruplet regimens or multi-drug combinations: 1 *Standard-risk* VRD, KRD, VCD,…,etc 2 *High-risk* Dara-VRD 3 *PCL or EMD* VDT-PACE • ASCT • Lenalidomide or Bortezomib maintenance therapy B-ASCT ineligible: • Bortezomib-based regimens: VRD or RD • Daratumumab, lenalidomide, dexamethasone: DRD • Alkylator-based regimens: VCD or VMP • Lenalidomide or Bortezomib maintenance therapy	N/A	• NCT05712083 (BCMA CAR-T Cell Therapy) • NCT05860036 (VRD + BCMA CAR-T Therapy) • NCT06140966 (Daratumumab, Carfilzomib-based Induction/Consolidation/Maintenance Therapy)
RRMM	Relapsed MM transpires after a phase of remission, whereas refractory MM implies a condition that either exhibits an unresponsiveness to treatment or regresses briefly after treatment concludes	• ASCT as salvage therapy if never done before • Consider time and initial drugs used before • *First Relapse* – Not Refractory to R: DRD or KRD – Refractory to R: KDD, Isa-KD, DPD, Isa-PD, KCD or KPD *• Second Relapse* Consider non refractory drug classes and Novels options	• CAR-T cell • Bispecific antibody therapy • Belantamab mafodotin • Selinexor • Isatuximab • Liposomal Doxorubicin • Venetoclax • Bendamustine • Ciltacabtagene autoleucel • Elranatamab-bcmm • Bispecific antibodies • Iberdomide • Modakafusp Alpha	• NCT06050512 (Mezigdomide, Ixazomib, Dexamethasone) • NCT05530421 (Selinexor, Venetoclax, Dexamethasone) • NCT05430945 (BCMA-targeted CAR-T Cells) • NCT05896228 (Iberdomide, Daratumumab, Carfilzomib, Dexamethasone) • NCT05704049 (Isatuximab, Carfilzomib, Dexamethasone)
PCL	• Conforms to the diagnostic criteria for multiple myeloma • The typical peripheral blood smear white blood cell differential count shows the presence of 5% or more plasma cells	*Transplant eligible* • Triplet (RVD, KRD) or RVD/KRD-PACE • ASCT or Allo SCT then Consolidation with PI/IMiD • Maintenance with PI/IMiD *Transplant ineligible* • Triplet RVD • Consolidation with PI/IMiD doublet/triplet • Maintenance with PI/IMiD	• Venetoclax • CAR-T cell • Bispecific Antibody Therapy • Antibody–Drug Conjugates	• NCT05870917 (anti-BCMA CAR-T) • NCT05979363 (anti-BCMA CAR-T)
EMD	• A biopsy identified a solitary lesion in soft tissue or bone containing clonal plasma cells • Presence or absence of clonal plasma cells in the bone marrow • Normal skeletal examination and MRI (or CT) of the spine and pelvis, except primary lesion • No CRAB	*Primary EMD* *Transplant eligible* • Triplet (RVD, KRD, PAD) • RVD-Daratumumab • VKDR-PACE • Followed by ASCT and Maintenance *Transplant ineligible* • RVD • Daratumumab-MPV • Daratumumab-VCD *Secondary EMD* • Polychemotherapy: DCEP • CAR-T cell therapy • Radiotherapy as palliative	For Secondary EMD • Pomalidomide–bortezomib–dexamethasone • Isatuximab–pomalidomide–dexamethasone • Selinexor–dexamethasone • Bendamustine–pomalidomide–dexamethasone	• NCT05201118 (BCMA-targeting CAR + Selinexor) • NCT05900882 (Selinexor, Bortezomib, Lenalidomide, Dexamethasone) • NCT06246162 (Mitoxantrone Hydrochloride Liposome Combination)

*BMPCs* Bone Marrow Plasma Cells, *CRAB* Calcium Renal Anemia Bone, *EMD* Extramedullary Disease, *MCP* Monoclonal Protein, *MDE* Myeloma Defining Events, *MGUS* Monoclonal Gammopathy of Undetermined Significance, *MM* Multiple Myeloma, *PCL* Plasma Cell Leukemia, *SMM* Smoldering Multiple Myeloma, *RRMM* Relapsed–Refractory Multiple Myeloma

## Multiple myeloma pathogenesis and chromosomal abnormalities

A sequence of genetic and microenvironmental changes promote the transformation of normal plasma cells into malignant MM. Plasma cells from MM and MGUS show early chromosomal abnormalities such as immunoglobulin heavy chain translocations or trisomies. Secondary translocations involving the MYC gene at 8q24, the MAF BZIP Transcription Factor B (MAFB) gene at 20q12, and the IRF4 gene at 6p25 are common in instances of MM, although they are uncommon in people with MGUS [11]. Mutations in RAS or FGFR3, dysregulation of MYC, deletion of p18, or loss of expression or mutation in TP53 are unique to MM and play an important role in tumor growth and the development of treatment resistance. In addition to molecular changes in plasma cells, the existence of abnormal connections between plasma cells and bone marrow, as well as the emergence of abnormal angiogenesis, are considered as key indications of disease progression [1].

Multiple myeloma has four main subtypes, with most people suffering falling into one of these categories. Trisomic MM, MM with the t (11; 14) translocation, MM with the t (4; 14) translocation, and MM with translocations including t (14; 16) or t (14; 20), often known as MAF MM, these are the most prevalent kinds of MM observed in clinical settings [12]. Certain chromosomal abnormalities, such as translocations involving chromosomes 4 and 14, 14 and 16, and 14 and 20, as well as deletion of the 17p area, gain of the 1q region, or mutation of the p53 gene, identify high-risk MM.

## Current therapy for multiple myeloma

Multiple myeloma care is constantly evolving, since a variety of pharmacological medicines have shown success in treating MM and are now available for clinical usage especially in 2015 after FDA approval of many antimyeloma drugs. As a result, there are several therapeutic regimens available for the management of MM in various clinical scenarios that include two or more of these pharmacologically active medications to control the sluggish proliferation or resilient MM cells by several ways [17].

There are two key criteria that determine the treatment approach to newly diagnosed patients with MM: their potential for autologous stem cell transplantation (ASCT) and the risk categorization procedure. Typically, characteristics such as age, performance status, and the presence of comorbidities determine appropriateness for ASCT. Contemporary therapy methods have shown the capacity to elicit dramatic responses, resulting in some patients achieving a minimum residual disease (MRD) negative status. While reaching a negative MRD status has been associated to improved progression-free survival (PFS) and overall survival (OS), it should be stressed that this is not the main goal of therapy [18].

A plethora of medication combinations have been developed to treat MM with each treatment demonstrating effectiveness via different mechanisms. To reduce possible toxicity, patients are often given a low-dose dexamethasone regimen (40 mg once weekly) throughout all treatment regimens [19]. Patients generally get three or four rounds of induction treatment before stem cell harvest. Following this, patients have the choice of undergoing frontline ASCT or continuing with induction therapy and postponing ASCT until the first relapse occurs. Bortezomib, lenalidomide, and dexamethasone (VRD) and daratumumab, lenalidomide, and dexamethasone (DRD) are the up-to-date standard of care for newly diagnosed MM [10] (Table 1).

In the context of individuals recently diagnosed with MM who are deemed ineligible for ASCT owing to factors such as advanced age or concurrent medical conditions, the treatment choices for first therapy are VRD (bortezomib, lenalidomide, and dexamethasone) and DRD (daratumumab, lenalidomide, and dexamethasone) [20].

## Relapsed–refractory multiple myeloma (RRMM)

The International Myeloma Work Group (IMWG) defines relapsed MM as the development of the disease after a favorable response to earlier therapy. Relapse might emerge as biochemical, radiological, or clinical event [21]. Progressive Disease may also be defined as the appearance of new soft tissue plasmacytomas or bone lesions or the expansion of any pre-existing one by at least 50%. Relapsed and refractory illness occurs when salvage therapy fails, or a patient deteriorates within 60 days of therapy [22].

Patients initially respond to therapy; nevertheless, as time passes, they acquire resistance. This is the fundamental challenge in the treatment of MM. Of the total number of patients starting first-line systemic treatment, 57% are on a proteasome inhibitor (PI), 23% are on an immunomodulatory agent (IMiD), and the remaining 20% on other regimens. The percentage of patients exposed to both PI and IMiD following the second, third, and fourth lines are 40.1%, 65.1%, and 86.0%, respectively. In any given year, over 10.0% of patients relapse and display resistance to both PIs and IMiDs [23].

Treatment in a relapse situation is aimed at managing the symptoms, stopping the development of organ damage, and returning the patient to a state of prolonged disease remission. When individuals with RRMM show treatment resistance, a different kind of regimen is started. While there is no wide agreement on prescription for the most effective therapy of RRMM, realistic treatment algorithms are devised based on available up-to-date data. Well-structured, long-term observational studies are required to verify the favorable effect of novel drugs and to find ways to prolong the quality of life of patients with MM [24].

## Challenges of multiple myeloma

Despite triplet and quadruplet induction regimens, ASCT, and maintenance treatment, myeloma remains incurable. Relapses occur at various phases of the progression of the disease [25]. It is critical to bear in mind that MM continues to be among the most financially burdensome forms of cancer [26, 27].

Extramedullary multiple myeloma (EMM) involvement or extramedullary disease (EMD) is defined as a metastatic stage of MM since it arises in the bone marrow. It can potentially manifest at any location in the body and might even appear before the primary diagnosis of multiple myeloma (MM). Current research indicates that 7% to 17% of MM cases present with EMD at the time of diagnosis. Moreover, as the disease progresses, an additional 6% to 20% of MM patients develop EMD, with the prevalence varying across different studies [14]. EMD is described as a clone's or subclone's ability to multiply independent of the bone marrow microenvironment [28]. Several genetic factors have been linked to EMD. These variables include 17p deletion, p53 nuclear expression, an elevated frequency of t (4; 14), and p53 deletion. Additionally the lack of CD56 expression, excessive MAFB expression, and excessive MYC expression are potential risk factors [28]. When presented at the time of diagnosis or recurrence, EMD boosts the complexity of existing MM and is associated with a poor clinical outcome, with an overall survival of fewer than 6 months. The clinical disorders linked with EMD vary significantly, and their management presents a major challenge [29].

Resistance to treatment is one of the main challenges of MM. Resistance may result from both MM cell internal and external causes. The internal causes include genetic and epigenetic changes that increase activity of drug efflux pumps, mutations or other changes in drug targets, and disruption of intracellular signaling networks that regulate processes such as apoptosis, autophagy, and DNA repair [30]. The external causes include communications with the bone marrow (BM) microenvironment, cell adherence to the extracellular matrix (ECM) etc. Bone marrow stromal cells (BMSCs) secrete soluble substances such as interleukin (IL)-6 and insulin-like growth factor (IGF)-1, these extrinsic factors trigger signal in MM cells, which eventually contributes to resistance to treatment [31]. A key strategy for the treatment of MM relapse involves using the proteasome inhibitor (PI) and thus resistance to PI represents a considerable therapeutic challenge. Although extensive research has been undertaken, these pathways have not been thoroughly verified in RRMM patient derived specimens, and hence their clinical importance remains uncertain [32].

Interesting recent research with single cell analysis revealed that gene signatures related to ribosome and translation are associated with a poor response of MM patients to bortezomib suggesting that ribosome dysregulation and alterations in mRNA translation activity contribute to PI resistance [33]. This prompted us to investigate relevance and potential of ribosomal pathways to this disease.

## Introduction to ribosome biogenesis

Human ribosome biogenesis involves a methodical assembly of 4 ribosomal RNAs and 80 ribosomal proteins. It begins inside the nucleolus with the formation of ribosomal RNA (rRNA). RNA polymerase I (Pol I) transcribes ribosomal DNA (rDNA) to generates 47S pre-rRNA, which undergoes intricately regulated splicing to produce 18S, 5.8S, and 28S rRNA molecules [34]. These rRNAs are secondarily modified by small nucleolar RNAs (snoRNAs). Ribosomal proteins (RPs) are imported from the cytosol into the nucleus, where they assemble with 5.8S and 28S rRNA molecules in addition to the 5S rRNA (transcribed from separate gene on chromosome one), to produce the 60S ribosomal subunit. The 18S rRNA molecule, following similar secondary modifications assembles with RPs to form the 40S ribosomal subunit [35]. Historically, the ribosomes were thought to be housekeeping entities for the purpose of protein synthesis rather than actively influencing the translational portfolio of the cells. Recent research has shown that the ribosome biogenesis is carefully regulated to support critical translation programs, independent of the cancer's genetic features [5]. Thus the progression of cancer is strongly dependent on changes in ribosome biogenesis [36]. Hyperactive ribosome biogenesis promotes uncontrolled cell proliferation and metastatic progression however mechanistic details are still emerging [37, 38].

Transcription factors such as MYC and OCA-B/BOB-1, that influence translation of cell fate determinants, are responsible for a considerable number of the strong and specific dependencies in MM, especially those that play an important role in defining plasma cell identity. The POU2AF1 gene, which encodes the OCA-B/BOB-1 protein, a coactivator protein involved in B cell transcription program and is expressed at all stages of B-cell maturation. Compared to normal plasma cells, it is more highly expressed in CD138+ plasma cells from patients with precursor illnesses (MGUS and SMM), as well as in symptomatic MM. BOB1 overexpression positively fosters de novo protein synthesis in MM cells through RNA Pol I-dependent transcription [39]. Oncogenic MYC plays a critical role in the progression of MM. MYC-induced genes are involved in a variety of biological functions, including cell growth, cell cycle control, energy production, anabolic metabolism, and DNA replication. The MYC protein has a direct beneficial effect on the transcription of genes encoding ribosomal proteins, ribosomal RNA, and translation initiation complex proteins [40]. MYC activation is seen in around 66% of newly diagnosed MM patients, and it is linked to poor clinical outcomes. Approximately 20 to 40% of patients may have MYC gene translocations [41, 42]. A key consequence of MYC activation is increased ribosome biogenesis, leading to an increased protein translation and increased biomass, which are characteristic of malignant cells [43, 44]. Manier and colleagues found that CMLD010509 inhibits the translation of a group of oncoproteins required for MM oncogenesis [5]. These oncoproteins comprise an oncogenic translation program. Furthermore, when examined in vivo, this medication had encouraging outcomes in a variety of mouse models, showing that targeting MYC in MM might be a feasible therapeutic approach that may help treat MM and other hematological malignancies [5]. These emerging findings in the context of our analysis of the MM patient data presented below highlight the importance of ribosome biogenesis in MM. Thus, understanding and exploiting ribosome biogenesis for MM targeted treatment approaches is important.

## Ribosome biogenesis, a novel therapeutic target

**Fig. 2:**
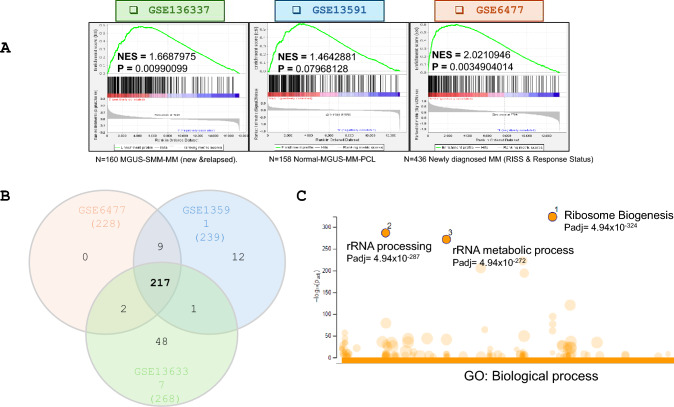
Ribosome biogenesis related gene expression is enriched in multiple myeloma patients: **A** Gene Set Enrichment Analysis (GSEA) was conducted on three multiple myeloma patient derived datasets: GSE136337 (N = 160), GSE13591 (N = 158), and GSE6477 (N = 436). The green curve represents enrichment profile. The normalized enrichment score (NES) and GSEA-derived P-value are indicated for each plot. **B** Venn diagram illustrating the overlap and uniqueness of the most significantly enriched genes identified through the GSEA of three multiple myeloma datasets: GSE6477, GSE13591 and GSE136337. Each circle represents a dataset, with GSE13591 in blue, GSE136337 in green, and GSE6477 in red. The intersections between the circles indicate genes that are commonly enriched across the datasets. This comparative analysis aids in identifying consistent molecular signatures across studies and provides insights into the complex genetic landscape of multiple myeloma. **C** The common set of 217 genes among the three datasets were used to perform Gene Ontology Analysis using g:profiler.™ to identify the associated biological processes. *Citations* Heberle H, Meirelles GV, da Silva FR, Telles GP, Minghim R (2015) InteractiVenn: a web-based tool for the analysis of sets through Venn diagrams. BMC Bioinform 16:169. 10.1186/s12859-015-0611-3

We queried three publicly available MM related datasets from the Gene Expression Omnibus (GEO) for ribosome biogenesis signatures (Fig. 2A). Our thought process was guided by well-established characteristic of the MM cells. That is, MM cells are highly secretory, and thus are highly dependent on protein translation. And that ribosome biogenesis is foundational to protein translation.

The data sets GSE13591 and GSE6477 were collected in 2009 and 2006 (last updated in 2018 and 2020 respectively). Both datasets contain the gene expression profiles of purified plasma cells (PCs) obtained from normal donors (N), monoclonal gammopathy of undetermined significance, 133 MM and 9 plasma cell leukemia (PCL) at diagnosis. The third MM dataset; GSE136337 (collected in 2019 and updated in 2020), contains gene expression data of 436 newly diagnosed multiple myeloma patients which were categorized into two groups based on the response status and the R-ISS (Revised International Staging System). Based on the response status, patients were classified into four groups complete response (CR), partial response (PR), stable disease (SD) and progressive disease (PD). Likewise, based on R-ISS they are classified as R-ISS I, R-ISS II and R-ISS III depending on the disease progression [45, 46].

We observed that all three data sets showed enrichment of ribosome biogenesis gene set [47]. In GSE136337 (N = 160 patients, P = 0.0099) and GSE6477 (N = 436 patients, P = 0.0034) ribosome biogenesis signature was significantly enriched. In GSE13591 (N = 158 patients, P = 0.079) we observed a noticeable trend (Fig. 2A). We then established 217 commonly enriched ribosome biogenesis genes across these three datasets as depicted in the Venn diagram (Fig. 2B). We then subjected the common set of 217 genes to gene ontology analysis using g:profiler™ and identified that genes associated with rRNA metabolism and rRNA processing were the main constituents of the 217 genes (Fig. 2C). This comparative analysis aided the identification of key constituents of ribosome biogenesis that change in multiple myeloma patients (Supplemental Table 1). We also observed that ribosome biogenesis is enhanced throughout the progression of MM from MGUS, SMM, and MM becoming an obvious feature in PCL. This is recognized not only within the clinical spectrum of MM, but it is also seen when patients are categorized into relapsed, progressing, and stable stages (Fig. 1B).

We then queried if ribosome biogenesis contributes to the progression of MM or resistance to treatment. Upon perusing through existing literature, we came across several notions that establish connections between ribosome biogenesis and MM advancement [5, 32, 48, 49]. Interesting recent research has revealed that polysome derived gene expression patterns, as well as translation initiation, were associated with a less-than-optimal response to the PI bortezomib in samples from MM patients. This shows that alterations in ribosome breakdown and in mRNA translation activity contribute to PI resistance [32, 33]. Thus, ribosome biogenesis may not influence MM progression but may also influence the drug resistance of MM.

## Progress on therapeutically targeting ribosome biogenesis

Ribosome formation may be restricted by a variety of anti-tumor medications as a by-product of their activity; however, there are few if any specialized drugs. Among all ribosome biosynthesis influencers, mammalian target of rapamycin (mTOR) has emerged as therapeutic candidates for multiple myeloma [32, 50]. mTOR, a cell's main nutritional sensor, promotes growth and proliferation. It regulates cellular energy sources for cell growth via several protein complexes. These complexes control protein translation to preserve the amino acid pool. Protein translation anomalies induced by mTOR pathway dysregulation are observed in different kinds of cancer. This pathway regulates protein translation via S6K1 and 4E-BP1. Crosstalk between mTOR, AKT, and PI3 kinase exacerbates dysfunction of this pathway [51–53]. According to recent studies pharmacological treatments that inhibit mTOR signaling and ribosome synthesis have the potential to slow the progression of cancer [50, 54, 55].

Ribosome biogenesis is regulated through nucleoli. The nucleoli are organized around the tandem repeats of rDNA on acrocentric chromosomes [56]. It is intuitive that chromatin dynamics plays an important role in ribosome biogenesis. SWI/SNF complex, is a prominent controller of chromatin dynamics. Bromodomain-containing protein 9 (BRD9), is a member of the bromodomain and extra-terminal (BET) protein family and a component of the SWI/SNF complex. BRD9 has bromodomain, which recognizes and binds to acetylated lysine residues on histone tails. This interaction influences chromatin structure and governs gene activation or repression [57]. The regulatory functions of BRD9 may be impaired by cancer, such as MM. Dysregulation may lead to uncontrolled growth of cancer cells since they often exploit the gene regulation function of BRD9. Ribosome biogenesis stimulation and BRD9 overexpression play critical roles in the development of multiple myeloma malignancy. Kenneth and his colleagues observed that decreasing BRD9 affected the formation of a transcription initiation complex and reduced ribosome biogenesis. This disruption influenced cell survival, indicating that BRD9 may be a viable therapeutic target for MM [58, 59]. In their recent investigation Kurata et al. showed that in Multiple Myeloma, targeting BRD9 disrupts ribosome biogenesis [58]. Thus, BRD9 inhibitors have the potential to become an important addition to the therapies for MM [48, 58].

The dysregulation of ribosome biogenesis in MM emphasizes its potential as a therapeutic target, creating a promising opportunity for the development of novel therapeutics. Thus, in MM targeting ribosome biogenesis has high value. While mTOR and BRD9 targeted approaches may allow ribosome biogenesis manipulation for MM, there are several other ways to target ribosome biogenesis [60]. Consequently, there is a significant opportunity to develop anti-tumor medications that specifically target genes implicated in the production of ribosomes [60]. For example, the major function of RNA polymerase (Pol I) is to transcribe ribosomal RNA (rRNA), which is an important step in the synthesis of ribosomes. This has prompted researchers to investigate specifically suppressing Pol I as a therapeutic approach [50, 61]. G-quadruplex structures are involved in rDNA transcription [62]. Various small-molecule ligands, such as cationic porphyrins and quindolines, can stabilize these structures. CX-5461, a highly selective inhibitor of RNA polymerase I that acts by attaching to the RNA polymerase I pre-initiation complex's selectivity factor 1 (SL1) and has demonstrated efficacy for in vivo applications is also a G-quadruplex stabilizer [63]. CX-5461 has shown remarkable effectiveness in various myeloma preclinical models, both in vitro and in vivo. CX-5461's therapeutic efficacy improves when MYC expression is increased, most likely owing to an increase in ribosome biosynthesis, which prepares cells that overexpress MYC for the repercussions of the nucleolar stress response [61]. CX-5461's powerful activity against human MM cells and PI-resistant mouse MM cells is possibly due to its dual mechanism of action, which includes suppressing ribosome synthesis and activating DDR (replication-dependent DNA damage response). It was evaluated in a Phase I clinical study for advanced hematologic malignancies and shown excellent tolerability. Most of the patients in the research had myeloma, and in 50% of cases, the best response was stable disease [64–66]. A derivate, CX-3543 (Quarfloxin) was introduced into clinical trials. Interestingly, Quarfloxin reduces MYC transcription and has successfully completed Phase I and II clinical trials in patients with advanced solid tumors and lymphomas (NCT00955786) and neuroendocrine/carcinoid tumors (NCT00780663), respectively [67]. Suppression of RNA pol I activity causes p53 pathway activation, cellular senescence, and autophagy, all of which increase cell death [68]. A phase I clinical study of CX-5461 as a monotherapy in 17 advanced patients of MM who had not responded to earlier regimens, showed that those with RRMM at the start of the trial had the best results, with three out of six patients obtaining stable condition after four to six treatment cycles [32]. CX-5461 clinical studies are now being conducted on subjects with advanced solid tumors (NCT02719977) and hematological cancer (ACTRN12613001061729) [68].

Another emerging lead is BMH-21 which possesses wide and potent antitumorigenic properties that effectively limit tumor growth. BMH-21 binds to DNA sequences rich in guanine and cytosine (GC-rich sequences), which are often found in genes associated to ribosomal DNA. It efficiently and swiftly inhibits the activity of RNA polymerase I (Pol I), the enzyme that transcribes ribosomal DNA [69]. It also causes the primary catalytic component of the Pol I complex to be degraded via the proteasome [70]. BMH-21 has been shown in hematological malignancies to effectively suppress rDNA transcription [70].

Ribosome biogenesis inhibition as an emerging treatment approach is not unique to MM but could extend to other solid malignancies. The combination of BMH21's ribosomal inhibition with the fibroblast growth factor receptor (FGFR) inhibitor Erdafitinib will be a potential therapy approach for glioma in the future [71]. Previous findings from our group demonstrate that Vismodegib and BMH-21 precisely target the dependence of radiation-exposed breast cancer cells, successfully preventing their dissemination to the lungs. Inhibiting RNA Pol I and Hedgehog activity in radiation therapy patients might deliver an important therapeutic benefit over existing breast cancer therapies [47].

## Conclusions

Multiple myeloma (MM) is a heterogeneous and genetically complex neoplasm with an approximate 5-year survival rate of 60% with recurring episodes of remission, treatment resistance and metastatic relapse. Considering their excessively high protein synthesis and processing rates, MM cells may be affected by an imbalance in protein homeostasis, in addition to a reduction in protein degradation which is generally achieved by polyubiquitination and proteasome transport. PIs are essential for frontline and RRMM patients and many patients face eventual resistance, which is a clinical obstacle. Recent research attributes suboptimal response of MM patients to PI (bortezomib), to disrupted ribosomal function and altered mRNA translation [32]. Tomasson et al. illustrated how ACA11 overexpression boosts MM cell line proliferation and changes nucleoli in both MM cell lines and patient MM tissues. Overexpression of ACA11 causes enhanced ROS-dependent ribosome biogenesis and protein synthesis, ultimately influencing MM chemotherapeutic response [49]. Overall focusing on ribosome biogenesis through RNA polymerase I inhibition seems to be promising based on the first-in-human study by Kylee et al. that used CX-5461 [32]. In conclusion ribosome biogenesis inhibition possibly through small inhibitors of RNA polymerase I, is a promising option to MM patients suffering from relapse or refractory state. The rapidly expanding area of MM and ribosome biogenesis certainly deserves quite a bit of curiosity from researchers.

## Supplementary Information

Below is the link to the electronic supplementary material.

Supplementary file1 (DOCX 24 KB)

## Data Availability

Details of all data generated or analyzed during this study are included in this article and its Supplementary Files. All authors have seen and approved the manuscript and consent publication.

## Abbreviations

ABC: ATP-Binding Cassette
ACA11: Autoinhibited Ca^2+^-ATPase 11
Allo SCT: Allogenic Stem Cell Transplant
ASCT: Autologous Stem Cell Transplantation
BCMA: B-Cell Maturation Antigen
BITE: Bi-Specific T-Cell Engagers
BOB-1: B Cell Transcriptional Coactivator POU2AF1
BM: Bone Marrow
BMSCs: Bone Marrow Stromal Cells
BMPCs: Bone Marrow Plasma Cells
BRD9: Bromodomain-containing protein 9
CAR-T: Chimeric Antigen Receptor T-Cell
CD: Cluster of Differentiation
CR: Complete Response
CRAB: Calcium Renal Anemia Bone
Daratumumab-MPV: Daratumumab, Melphalan, Prednisolone, Bortezomib
Dara-VRd: Daratumumab Bortezomib, Lenalidomide, Dexamethasone
Dara-VTD: Daratumumab Bortezomib, Thalidomide, Dexamethasone
DCEP: Dexamethasone, Cyclophosphamide, Etoposide, Cisplatin
DDR: DNA Damage Response
DNA: Deoxyribonucleic Acid
DPD: Daratumumab, Pomalidomide, Dexamethasone
DRD: Daratumumab, Lenalidomide, Dexamethasone
ECM: Extracellular Matrix
EMD: Extramedullary Disease
FDA: Food and Drug Administration
FGFR3: Fibroblast Growth Factor Receptor 3
FISH: Fluorescent In Situ Hybridization
FLCR: Free Light Chain Ratio
GEO: Gene Expression Omnibus
GEP: Gene Expression Profiling
GSE: Gene Set Enrichment
GSEA: Gene Set Enrichment Analysis
IgA: Immunoglobulin A
IgG: Immunoglobulin G
IMiD: Immunomodulatory Agent
IMWG: International Myeloma Work Group
IRF4: Interferon Regulatory Factor 4
Isa-KD: Isatuximab, Carfilzomib, Dexamethasone
Isa-PD: Isatuximab, Pomalidomide, Dexamethasone
KCD: Carfilzomib, Cyclophosphamide Dexamethasone
KDD: Carfilzomib, Daratumumab, Dexamethasone
KPD: Carfilzomib, Pomalidomide, Dexamethasone
KRD: Carfilzomib, Lenalidomide, Dexamethasone
KRD-PACE: Carfilzomib, Lenalidomide, Dexamethasone. Cisplatin, Doxorubicin, Cyclophosphamide, and Etoposide
MAFB: MAF BZIP Transcription Factor B
MCP: Monoclonal Protein
MDE: Myeloma Defining Events
MGUS: Monoclonal Gammopathy of Undetermined Significance
MM: Multiple Myeloma
MRD: Minimum Residual Disease
MRI: Magnetic Resonance Imaging
mRNA: Messenger Ribonucleic Acid
mTOR: Mammalian Target of Rapamycin
MYC: Myelocytomatosis Oncogene
OS: Overall Survival
PAD: Bortezomib, Doxorubicin, Dexamethasone
PCL: Plasma Cell Leukemia
PD: Progressive Disease
PET/CT: Positron Emission Tomography/Computed Tomography
PFS: Progression-Free Survival
PI: Proteasome Inhibitor
PR: Partial Response
RAS: Rat Sarcoma
rDNA: Ribosomal Deoxyribonucleic Acid
R-ISS: Revised International Staging System
RNA pol I: Ribonucleic Acid Polymerase I
RNA-Seq: RNA Sequencing
RPs: Ribosomal Proteins
RRMM: Relapsed–Refractory Multiple Myeloma
rRNA: Ribosomal Ribonucleic Acid
SD: Stable Disease
SIFE: Serum Immunofixation
SMM: Smoldering Multiple Myeloma
snoRNAs: Small Nucleolar Ribonucleic Acid
SPEP: Serum Protein Electrophoresis
SWI/SNF: SWItch/Sucrose Non-Fermentable, an ATP-Dependent Chromatin Remodeling Complexes
t: Translocation
TP53: Tumor Suppressor Gene:
V(K)RD-PACE: Bortezomib (Carfilzomib), Lenalidomide, Dexamethasone, Cisplatin, Doxorubicin, Cyclophosphamide and Etoposide
VCD: Bortezomib, Cyclophosphamide Dexamethasone
VDT-PACE: Bortezomib, Dexamethasone, Thalidomide, Cisplatin, Doxorubicin, Cyclophosphamide and Etoposide
VMP: Bortezomib, Melphalan, Prednisolone
VRd: Bortezomib, Lenalidomide, Dexamethasone
VTD: Bortezomib, Thalidomide, Dexamethasone
WB-CT: Whole-Body Computed Tomography

## References

[1] Palumbo A, Anderson K (2011) Multiple myeloma. N Engl J Med 364(11):1046–1060 21410373 10.1056/NEJMra1011442

[2] McCurdy A, Seow H, Pond GP, Gayowsky A, Chakraborty R, Visram A et al (2023) Cancer-specific mortality in multiple myeloma: a population-based retrospective cohort study. Haematologica 108(12):3384–3391 37439357 10.3324/haematol.2023.282905 PMC10690919

[3] Zhou L, Yu Q, Wei G, Wang L, Huang Y, Hu K et al (2021) Measuring the global, regional, and national burden of multiple myeloma from 1990 to 2019. BMC Cancer 21(1):606 34034700 10.1186/s12885-021-08280-y PMC8152089

[4] Andrade CLB, Ferreira MV, Alencar BM, Junior AMA, Lopes TJS, dos Santos AS et al (2024) Enhancing diagnostic accuracy of multiple myeloma through ML-driven analysis of hematological slides: new dataset and identification model to support hematologists. Sci Rep 14(1):11176 38750071 10.1038/s41598-024-61420-9 PMC11096332

[5] Manier S, Huynh D, Shen YJ, Zhou J, Yusufzai T, Salem KZ et al (2017) Inhibiting the oncogenic translation program is an effective therapeutic strategy in multiple myeloma. Sci Transl Med 9(389):eaal2668 28490664 10.1126/scitranslmed.aal2668 PMC5718051

[6] Fend F, Dogan A, Cook JR (2023) Plasma cell neoplasms and related entities-evolution in diagnosis and classification. Virchows Arch 482(1):163–177 36414803 10.1007/s00428-022-03431-3 PMC9852202

[7] Madhira BR, Konala VM, Adapa S, Naramala S, Ravella PM, Parikh K et al (2020) Recent advances in the management of smoldering multiple myeloma. World J Oncol 11(2):45–54 32284772 10.14740/wjon1245 PMC7141158

[8] Hussain M, Yellapragada S, Al HS (2023) Differential diagnosis and therapeutic advances in multiple myeloma: a review article. Blood Lymphat Cancer 13:33–57 37731771 10.2147/BLCTT.S272703 PMC10508231

[9] Cowan AJ, Green DJ, Kwok M, Lee S, Coffey DG, Holmberg LA et al (2022) Diagnosis and management of multiple myeloma: a review. JAMA 327(5):464–477 35103762 10.1001/jama.2022.0003

[10] Rajkumar SV (2022) Multiple myeloma: 2022 update on diagnosis, risk stratification, and management. Am J Hematol 97(8):1086–1107 35560063 10.1002/ajh.26590 PMC9387011

[11] Cardona-Benavides IJ, de Ramón C, Gutiérrez NC (2021) Genetic abnormalities in multiple myeloma: prognostic and therapeutic implications. Cells 10(2):336 33562668 10.3390/cells10020336 PMC7914805

[12] Bal S, Kumar SK, Fonseca R, Gay F, Hungria VT, Dogan A et al (2022) Multiple myeloma with t(11; 14): unique biology and evolving landscape. Am J Cancer Res 12(7):2950–2965 35968339 PMC9360221

[13] Tuazon SA, Holmberg LA, Nadeem O, Richardson PG (2021) A clinical perspective on plasma cell leukemia; current status and future directions. Blood Cancer J 11(2):23 33563906 10.1038/s41408-021-00414-6 PMC7873074

[14] Li Y, Sun Z, Qu X (2022) Advances in the treatment of extramedullary disease in multiple myeloma. Transl Oncol 22:101465 35679743 10.1016/j.tranon.2022.101465 PMC9178475

[15] NCC Center (2024) Multiple myeloma 2024 (version 3.2024). https://www.nccn.org/professionals/physician_gls/pdf/myeloma.pdf

[16] Annamaria G, Kenneth CA (2020) Multiple myeloma: the (r)evolution of current therapy and a glance into future. Haematologica 105(10):2358–2367 33054076 10.3324/haematol.2020.247015 PMC7556665

[17] Mikhael J, Ismaila N, Cheung MC, Costello C, Dhodapkar MV, Kumar S et al (2019) Treatment of multiple myeloma: ASCO and CCO joint clinical practice guideline. J Clin Oncol 37(14):1228–1263 30932732 10.1200/JCO.18.02096

[18] Rajkumar SV, Kumar S (2020) Multiple myeloma current treatment algorithms. Blood Cancer J 10(9):94 32989217 10.1038/s41408-020-00359-2 PMC7523011

[19] Burwick N, Sharma S (2019) Glucocorticoids in multiple myeloma: past, present, and future. Ann Hematol 98(1):19–28 30073393 10.1007/s00277-018-3465-8

[20] Costello CL (2022) Newly diagnosed multiple myeloma: making sense of the menu. Hematology 2022(1):539–550 36485145 10.1182/hematology.2022000404 PMC9820388

[21] Dima D, Ullah F, Mazzoni S, Williams L, Faiman B, Kurkowski A et al (2023) Management of relapsed–refractory multiple myeloma in the era of advanced therapies: evidence-based recommendations for routine clinical practice. Cancers 15(7):2160 37046821 10.3390/cancers15072160 PMC10093129

[22] Kumar S, Paiva B, Anderson KC, Durie B, Landgren O, Moreau P et al (2016) International Myeloma Working Group consensus criteria for response and minimal residual disease assessment in multiple myeloma. Lancet Oncol 17(8):e328–e346 27511158 10.1016/S1470-2045(16)30206-6

[23] Gatopoulou X, Bardenheuer K, Van Hoorenbeeck S, Kempel A (2016) PCN42—treatment patterns of relapsed and refractory multiple myeloma in Europe (EU-28). Value Health 19(7):A347–A766

[24] Lee JH, Kim S-H (2020) Treatment of relapsed and refractory multiple myeloma. Blood Res 55:S43–S53 32719176 10.5045/br.2020.S008 PMC7386890

[25] Bhatt P, Kloock C, Comenzo R (2023) Relapsed/refractory multiple myeloma: a review of available therapies and clinical scenarios encountered in myeloma relapse. Curr Oncol 30(2):2322–2347 36826140 10.3390/curroncol30020179 PMC9954856

[26] Das S, Juliana N, Yazit NAA, Azmani S, Abu IF (2022) Multiple myeloma: challenges encountered and future options for better treatment. Int J Mol Sci 23(3):1649 35163567 10.3390/ijms23031649 PMC8836148

[27] Mateos M-V, Nooka AK, Larson SM (2022) Moving toward a cure for myeloma. Am Soc Clin Oncol Educ Book 42:643–654 10.1200/EDBK_349603 35623025

[28] Bladé J, Beksac M, Caers J, Jurczyszyn A, von Lilienfeld-Toal M, Moreau P et al (2022) Extramedullary disease in multiple myeloma: a systematic literature review. Blood Cancer J 12(3):45 35314675 10.1038/s41408-022-00643-3 PMC8938478

[29] Touzeau C, Moreau P (2016) How I treat extramedullary myeloma. Blood 127(8):971–976 26679866 10.1182/blood-2015-07-635383

[30] Pinto V, Bergantim R, Caires HR, Seca H, Guimarães JE, Vasconcelos MH (2020) Multiple myeloma: available therapies and causes of drug resistance. Cancers 12(2):407 32050631 10.3390/cancers12020407 PMC7072128

[31] Solimando AG, Malerba E, Leone P, Prete M, Terragna C, Cavo M et al (2022) Drug resistance in multiple myeloma: soldiers and weapons in the bone marrow niche. Front Oncol 12:973836 36212502 10.3389/fonc.2022.973836 PMC9533079

[32] Maclachlan KH, Gitareja K, Kang J, Cuddihy A, Cao Y, Hein N et al (2024) Targeting the ribosome to treat multiple myeloma. Mol Ther Oncol 32(1):200771 38596309 10.1016/j.omton.2024.200771 PMC10905045

[33] Jung S-H, Park S-S, Lim J-Y, Sohn SY, Kim NY, Kim D et al (2022) Single-cell analysis of multiple myelomas refines the molecular features of bortezomib treatment responsiveness. Exp Mol Med 54(11):1967–1978 36380017 10.1038/s12276-022-00884-z PMC9723182

[34] Ni C, Buszczak M (2023) The homeostatic regulation of ribosome biogenesis. Semin Cell Dev Biol 136:13–26 35440410 10.1016/j.semcdb.2022.03.043 PMC9569395

[35] Jiao L, Liu Y, Yu X-Y, Pan X, Zhang Y, Tu J et al (2023) Ribosome biogenesis in disease: new players and therapeutic targets. Signal Transduct Target Ther 8(1):15 36617563 10.1038/s41392-022-01285-4 PMC9826790

[36] Turi Z, Lacey M, Mistrik M, Moudry P (2019) Impaired ribosome biogenesis: mechanisms and relevance to cancer and aging. Aging (Albany NY) 11(8):2512–2540 31026227 10.18632/aging.101922 PMC6520011

[37] Elhamamsy AR, Metge BJ, Alsheikh HA, Shevde LA, Samant RS (2022) Ribosome biogenesis: a central player in cancer metastasis and therapeutic resistance. Cancer Res 82(13):2344–2353 35303060 10.1158/0008-5472.CAN-21-4087 PMC9256764

[38] Ban Y, Zou Y, Liu Y, Lee SB, Bednarczyk RB, Sheng J et al (2024) Targeting ribosome biogenesis as a novel therapeutic approach to overcome EMT-related chemoresistance in breast cancer. bioRxiv 10.7554/eLife.89486 PMC11390108 39259576

[39] Chyra Z, Samur MK, Aktas-Samur A, Yao Y, Derebail S, Perini T et al (2021) B cell transcriptional coactivator POU2AF1 (BOB-1) is an early transcription factor modulating the protein synthesis and ribosomal biogenesis in multiple myeloma: with therapeutic implication. Blood 138:267033945616

[40] Raimondi V, Iannozzi NT, Burroughs-Garcìa J, Toscani D, Storti P, Giuliani N (2022) A personalized molecular approach in multiple myeloma: the possible use of RAF/RAS/MEK/ERK and BCL-2 inhibitors. Explor Target Antitumor Ther 3(4):463–479 36071980 10.37349/etat.2022.00095 PMC9446161

[41] Chng WJ, Huang GF, Chung TH, Ng SB, Gonzalez-Paz N, Troska-Price T et al (2011) Clinical and biological implications of MYC activation: a common difference between MGUS and newly diagnosed multiple myeloma. Leukemia 25(6):1026–1035 21468039 10.1038/leu.2011.53 PMC3432644

[42] Affer M, Chesi M, Chen WD, Keats JJ, Demchenko YN, Tamizhmani K et al (2014) Promiscuous MYC locus rearrangements hijack enhancers but mostly super-enhancers to dysregulate MYC expression in multiple myeloma. Leukemia 28(8):1725–1735 24518206 10.1038/leu.2014.70 PMC4126852

[43] Ahmadi SE, Rahimi S, Zarandi B, Chegeni R, Safa M (2021) MYC: a multipurpose oncogene with prognostic and therapeutic implications in blood malignancies. J Hematol Oncol 14(1):121 34372899 10.1186/s13045-021-01111-4 PMC8351444

[44] Zielke N, Vähärautio A, Liu J, Kivioja T, Taipale J (2022) Upregulation of ribosome biogenesis via canonical E-boxes is required for Myc-driven proliferation. Dev Cell 57(8):1024–36.e5 35472319 10.1016/j.devcel.2022.03.018

[45] Subramanian A, Tamayo P, Mootha VK, Mukherjee S, Ebert BL, Gillette MA et al (2005) Gene set enrichment analysis: a knowledge-based approach for interpreting genome-wide expression profiles. Proc Natl Acad Sci USA 102(43):15545–15550 16199517 10.1073/pnas.0506580102 PMC1239896

[46] Mootha VK, Lindgren CM, Eriksson K-F, Subramanian A, Sihag S, Lehar J et al (2003) PGC-1α-responsive genes involved in oxidative phosphorylation are coordinately downregulated in human diabetes. Nat Genet 34(3):267–273 12808457 10.1038/ng1180

[47] Metge BJ, Alsheikh HA, Chen D, Elhamamsy AR, Hinshaw DC, Chen BR et al (2023) Ribosome biosynthesis and Hedgehog activity are cooperative actionable signaling mechanisms in breast cancer following radiotherapy. NPJ Precis Oncol 7(1):61 37380890 10.1038/s41698-023-00410-y PMC10307872

[48] Kurata K, Samur MK, Liow P, Wen K, Yamamoto L, Liu J et al (2022) BRD9 is essential for ribosome biogenesis and the survival of multiple myeloma cells. Blood 140(Supplement 1):596–597

[49] Oliveira V, Mahajan N, Bates ML, Tripathi C, Kim KQ, Zaher HS et al (2019) The snoRNA target of t(4; 14) in multiple myeloma regulates ribosome biogenesis. FASEB Bioadv 1(7):404–414 32095781 10.1096/fba.2018-00075 PMC6996358

[50] Wang Y, Vandewalle N, De Veirman K, Vanderkerken K, Menu E, De Bruyne E (2024) Targeting mTOR signaling pathways in multiple myeloma: biology and implication for therapy. Cell Commun Signal 22(1):320 38862983 10.1186/s12964-024-01699-3 PMC11165851

[51] Showkat M, Beigh MA, Andrabi KI (2014) mTOR signaling in protein translation regulation: implications in cancer genesis and therapeutic interventions. Mol Biol Int 2014(1):686984 25505994 10.1155/2014/686984 PMC4258317

[52] Lu Y, Wang S, Jiao Y (2023) The effects of deregulated ribosomal biogenesis in cancer. Biomolecules 13(11):1593 38002277 10.3390/biom13111593 PMC10669593

[53] Panwar V, Singh A, Bhatt M, Tonk RK, Azizov S, Raza AS et al (2023) Multifaceted role of mTOR (mammalian target of rapamycin) signaling pathway in human health and disease. Signal Transduct Target Ther 8(1):375 37779156 10.1038/s41392-023-01608-z PMC10543444

[54] Bouyahya A, El Allam A, Aboulaghras S, Bakrim S, El Menyiy N, Alshahrani MM et al (2022) Targeting mTOR as a cancer therapy: recent advances in natural bioactive compounds and immunotherapy. Cancers (Basel) 14(22):5520 36428613 10.3390/cancers14225520 PMC9688668

[55] Gentilella A, Kozma SC, Thomas G (2015) A liaison between mTOR signaling, ribosome biogenesis and cancer. Biochim Biophys Acta 1849(7):812–820 25735853 10.1016/j.bbagrm.2015.02.005 PMC4766360

[56] Weeks SE, Metge BJ, Samant RS (2019) The nucleolus: a central response hub for the stressors that drive cancer progression. Cell Mol Life Sci 76(22):4511–4524 31338556 10.1007/s00018-019-03231-0 PMC6841648

[57] Colarusso E, Chini MG, Bifulco G, Lauro G, Giordano A (2024) Identification and development of BRD9 chemical probes. Pharmaceuticals 17(3):392 38543178 10.3390/ph17030392 PMC10976250

[58] Kurata K, Samur MK, Liow P, Wen K, Yamamoto L, Liu J et al (2023) BRD9 degradation disrupts ribosome biogenesis in multiple myeloma. Clin Cancer Res 29(9):1807–1821 36780189 10.1158/1078-0432.CCR-22-3668 PMC10150249

[59] Chowdhury B, Garg S, Ni W, Sattler M, Sanchez D, Meng C et al (2024) Synergy between BRD9- and IKZF3-targeting as a therapeutic strategy for multiple myeloma. Cancers 16(7):1319 38610997 10.3390/cancers16071319 PMC11010819

[60] Zang Y, Ran X, Yuan J, Wu H, Wang Y, Li H et al (2024) Genomic hallmarks and therapeutic targets of ribosome biogenesis in cancer. Brief Bioinform 25(2):bbae023 38343327 10.1093/bib/bbae023 PMC10859687

[61] Lee HC, Wang H, Baladandayuthapani V, Lin H, He J, Jones RJ et al (2017) RNA polymerase I inhibition with CX-5461 as a novel therapeutic strategy to target MYC in multiple myeloma. Br J Haematol 177(1):80–94 28369725 10.1111/bjh.14525 PMC5695568

[62] Datta A, Pollock KJ, Kormuth KA, Brosh RM Jr (2021) G-quadruplex assembly by ribosomal DNA: emerging roles in disease pathogenesis and cancer biology. Cytogenet Genome Res 161(6–7):285–296 34469893 10.1159/000516394 PMC8455414

[63] Xu H, Di Antonio M, McKinney S, Mathew V, Ho B, O’Neil NJ et al (2017) CX-5461 is a DNA G-quadruplex stabilizer with selective lethality in BRCA1/2 deficient tumours. Nat Commun 8:14432 28211448 10.1038/ncomms14432 PMC5321743

[64] Martínez-Martín S, Soucek L (2021) MYC inhibitors in multiple myeloma. Cancer Drug Resist 4(4):842–865 35582389 10.20517/cdr.2021.55 PMC8992455

[65] Pelletier J, Thomas G, Volarević S (2018) Ribosome biogenesis in cancer: new players and therapeutic avenues. Nat Rev Cancer 18(1):51–63 29192214 10.1038/nrc.2017.104

[66] Wang M, Vulcano S, Xu C, Xie R, Peng W, Wang J et al (2024) Potentials of ribosomopathy gene as pharmaceutical targets for cancer treatment. J Pharm Anal 14(3):308–320 38618250 10.1016/j.jpha.2023.10.001 PMC11010632

[67] Xu H, Hurley LH (2022) A first-in-class clinical G-quadruplex-targeting drug. The bench-to-bedside translation of the fluoroquinolone QQ58 to CX-5461 (Pidnarulex). Bioorg Med Chem Lett 77:129016 36195286 10.1016/j.bmcl.2022.129016

[68] Hald ØH, Olsen L, Gallo-Oller G, Elfman LHM, Løkke C, Kogner P et al (2019) Inhibitors of ribosome biogenesis repress the growth of MYCN-amplified neuroblastoma. Oncogene 38(15):2800–2813 30542116 10.1038/s41388-018-0611-7 PMC6484764

[69] Peltonen K, Colis L, Liu H, Trivedi R, Moubarek Michael S, Moore Henna M et al (2014) A targeting modality for destruction of RNA polymerase I that possesses anticancer activity. Cancer Cell 25(1):77–90 24434211 10.1016/j.ccr.2013.12.009 PMC3930145

[70] Derenzini E, Rossi A, Treré D (2018) Treating hematological malignancies with drugs inhibiting ribosome biogenesis: when and why. J Hematol Oncol 11(1):75 29855342 10.1186/s13045-018-0609-1 PMC5984324

[71] Zisi A, Kanellis DC, Moussaud S, Karlsson I, Carén H, Bräutigam L et al (2022) Small molecule-mediated disruption of ribosome biogenesis synergizes with FGFR inhibitors to suppress glioma cell growth. Neurooncology 25(6):1058–1072 10.1093/neuonc/noac286 PMC10237440 36583853

