# Supplementary material for: Multiple myeloma: clinical characteristics, current therapies and emerging innovative treatments targeting ribosome biogenesis dynamics
Source: Clin Exp Metastasis. 2024 Aug 20;41(6):829–42. doi: 10.1007/s10585-024-10305-2 (PMC11607061; doi:10.1007/s10585-024-10305-2)
Supplement: Supplementary file 1 — Supplementary file1 (DOCX 24 KB) [file 10585_2024_10305_MOESM1_ESM.docx]

**Supplemental Table 1:**

|  | **List of common genes from ribosome biogenesis signature that affects MM** |
| --- | --- |
| 1 | ABCE1 |
| 2 | ABT1 |
| 3 | BMS1 |
| 4 | BRIX1 |
| 5 | BUD23 |
| 6 | BYSL |
| 7 | C1D |
| 8 | C1QBP |
| 9 | CHD7 |
| 10 | CUL4A |
| 11 | CUL4B |
| 12 | DCAF13 |
| 13 | DDX10 |
| 14 | DDX17 |
| 15 | DDX18 |
| 16 | DDX21 |
| 17 | DDX27 |
| 18 | DDX28 |
| 19 | DDX31 |
| 20 | DDX3X |
| 21 | DDX49 |
| 22 | DDX51 |
| 23 | DDX54 |
| 24 | DDX56 |
| 25 | DHX29 |
| 26 | DHX30 |
| 27 | DIMT1 |
| 28 | DIS3 |
| 29 | DROSHA |
| 30 | EFL1 |
| 31 | EIF4A3 |
| 32 | EIF6 |
| 33 | EMG1 |
| 34 | ERAL1 |
| 35 | ERCC2 |
| 36 | ERI2 |
| 37 | ERI3 |
| 38 | ESF1 |
| 39 | EXOSC1 |
| 40 | EXOSC10 |
| 41 | EXOSC2 |
| 42 | EXOSC4 |
| 43 | EXOSC5 |
| 44 | EXOSC7 |
| 45 | EXOSC8 |
| 46 | EXOSC9 |
| 47 | FASTKD2 |
| 48 | FCF1 |
| 49 | FTSJ3 |
| 50 | GAR1 |
| 51 | GEMIN4 |
| 52 | GLUL |
| 53 | GNL2 |
| 54 | GNL3L |
| 55 | GPATCH4 |
| 56 | GTF2H5 |
| 57 | GTF3A |
| 58 | GTPBP4 |
| 59 | HEATR1 |
| 60 | HEATR3 |
| 61 | IMP3 |
| 62 | IMP4 |
| 63 | ISG20 |
| 64 | ISG20L2 |
| 65 | KAT2B |
| 66 | KRI1 |
| 67 | KRR1 |
| 68 | LAS1L |
| 69 | LSG1 |
| 70 | LSM6 |
| 71 | MAK16 |
| 72 | MDN1 |
| 73 | METTL17 |
| 74 | METTL18 |
| 75 | METTL25B |
| 76 | METTL5 |
| 77 | MPHOSPH1 |
| 78 | MPHOSPH6 |
| 79 | MRM2 |
| 80 | MRM3 |
| 81 | MRPL20 |
| 82 | MRPL22 |
| 83 | MRPS11 |
| 84 | MRPS2 |
| 85 | MRPS7 |
| 86 | MRTO4 |
| 87 | MTERF3 |
| 88 | MTERF4 |
| 89 | MTREX |
| 90 | MYBBP1A |
| 91 | NAT10 |
| 92 | NGDN |
| 93 | NGRN |
| 94 | NHP2 |
| 95 | NIP7 |
| 96 | NLE1 |
| 97 | NMD3 |
| 98 | NOC2L |
| 99 | NOC4L |
| 100 | NOL10 |
| 101 | NOL11 |
| 102 | NOL6 |
| 103 | NOL8 |
| 104 | NOL9 |
| 105 | NOLC1 |
| 106 | NOP10 |
| 107 | NOP14 |
| 108 | NOP16 |
| 109 | NOP2 |
| 110 | NPM1 |
| 111 | NPM3 |
| 112 | NSA2 |
| 113 | NSUN3 |
| 114 | NSUN5 |
| 115 | NUP88 |
| 116 | NVL |
| 117 | PA2G4 |
| 118 | PAK1IP1 |
| 119 | PDCD11 |
| 120 | PES1 |
| 121 | PIH1D1 |
| 122 | PIN4 |
| 123 | POP4 |
| 124 | POP5 |
| 125 | POP7 |
| 126 | PRKDC |
| 127 | PTEN |
| 128 | PWP1 |
| 129 | RAN |
| 130 | RBFA |
| 131 | RCL1 |
| 132 | REXO4 |
| 133 | RIOK2 |
| 134 | RIOK3 |
| 135 | RIOX2 |
| 136 | RNASEL |
| 137 | RPF1 |
| 138 | RPL10A |
| 139 | RPL10L |
| 140 | RPL11 |
| 141 | RPL14 |
| 142 | RPL23A |
| 143 | RPL24 |
| 144 | RPL26L1 |
| 145 | RPL27 |
| 146 | RPL3 |
| 147 | RPL35 |
| 148 | RPL35A |
| 149 | RPL38 |
| 150 | RPL3L |
| 151 | RPL7 |
| 152 | RPLP0 |
| 153 | RPP25 |
| 154 | RPP30 |
| 155 | RPP38 |
| 156 | RPP40 |
| 157 | RPS14 |
| 158 | RPS15 |
| 159 | RPS16 |
| 160 | RPS19 |
| 161 | RPS21 |
| 162 | RPS24 |
| 163 | RPS27 |
| 164 | RPS27L |
| 165 | RPS28 |
| 166 | RPS5 |
| 167 | RPS6 |
| 168 | RPS7 |
| 169 | RPS8 |
| 170 | RPUSD2 |
| 171 | RRP1 |
| 172 | RRP12 |
| 173 | RRP15 |
| 174 | RRP1B |
| 175 | RRP7A |
| 176 | RRP8 |
| 177 | RRP9 |
| 178 | RRS1 |
| 179 | RSL1D1 |
| 180 | RSL24D1 |
| 181 | SART1 |
| 182 | SDAD1 |
| 183 | SHQ1 |
| 184 | SIRT7 |
| 185 | SNU13 |
| 186 | SUV39H1 |
| 187 | TBL3 |
| 188 | TFB1M |
| 189 | TFB2M |
| 190 | TRMT112 |
| 191 | TRMT61B |
| 192 | TSC1 |
| 193 | TSR1 |
| 194 | TSR2 |
| 195 | TSR3 |
| 196 | URB1 |
| 197 | URB2 |
| 198 | USP36 |
| 199 | UTP11 |
| 200 | UTP14A |
| 201 | UTP18 |
| 202 | UTP20 |
| 203 | UTP25 |
| 204 | UTP3 |
| 205 | UTP6 |
| 206 | WBP11 |
| 207 | WDR12 |
| 208 | WDR18 |
| 209 | WDR3 |
| 210 | WDR43 |
| 211 | WDR55 |
| 212 | WDR74 |
| 213 | XPO1 |
| 214 | XRCC5 |
| 215 | YTHDF2 |
| 216 | ZCCHC4 |
| 217 | ZNHIT6 |
|  |  |
